# The 1% gift to humanity: The Human Genome Project II

**DOI:** 10.1038/s41422-024-01026-y

**Published:** 2024-09-11

**Authors:** Weibin Liu, Yan Li, George P. Patrinos, Shuhua Xu, Meow-Keong Thong, Zhengming Chen, Francis P. Crawley, Liming Li, Perihan Elif Ekmekci, Radoje Drmanac, Weiyang Cheong, Robert Benamouzig, Quan Nguyen, Pavel Volchkov, Juergen K. V. Reichardt, Piero Carninci, Partha Majumder, Xin Jin, George Church, Jian Wang, Xun Xu

**Affiliations:** 1 BGI, Shenzhen, China; 2 BGI Research, Shenzhen, China; 3 Department of Pharmacy, School of Health Sciences, University of Patras, Patras, Greece; 4 Center for Evolutionary Biology, School of Life Sciences, Fudan University, Shanghai, China; 5 Department of Population Medicine, M. Kandiah Faculty of Medicine and Health Sciences, Universiti Tunku Abdul Rahman, Selangor, Malaysia; 6 Genetic & Metabolism Unit, Department of Paediatrics, Faculty of Medicine, University of Malaya, Kuala Lumpur, Malaysia; 7 Clinical Trial Service Unit & Epidemiological Studies Unit, Nuffield Department of Population Health, University of Oxford, Oxford, UK; 8 CODATA International Data Policy Committee (IDPC) and Strategic Initiative for Developing Capacity in Ethical Review-Europe (SIDCER), Leuven, Belgium; 9 Department of Epidemiology & Biostatistics, School of Public Health, Peking University, Beijing, China; 10 Peking University Center for Public Health and Epidemic Preparedness & Response, Beijing, China; 11 Key Laboratory of Epidemiology of Major Diseases (Peking University), Ministry of Education, Beijing, China; 12 Department of History of Medicine and Ethics Bioethics/WMA Cooperation Center, School of Medicine, TOBB University of Economics and Technology, and CODATA International Data Policy Committee, Ankara, Turkey; 13 MGI, San Jose, CA USA; 14 Provost Office, Singapore Management University, Singapore, Singapore; 15 Department of Gastroenterology and Digestive Oncology, AP-HP Avicenne Hospital, Sorbonne Paris Nord University, Bobigny, France; 16 Hanoi Medical University, Hanoi, Vietnam; 17 University of Queensland, Queensland, Australia; 18 Genome Engineering Laboratory, Moscow Institute of Physics and Technology, Dolgoprudniy, Russian Federation; 19 Australian Institute of Tropical Health and Medicine, James Cook University, Smithfield, QLD Australia; 20 Human Technopole, Milan, Italy; 21 RIKEN Center for Integrative Medical Sciences, Yokohama, Japan; 22 John C. Martin Centre for Liver Research and Innovations, Kolkata, India; 23 Department of Genetics, Harvard Medical School, Boston, MA USA

**Keywords:** Genetic variation, Bioinformatics

Upon announcing the completion of the Human Genome Project (HGP) in 2003, scientific leaders envisaged the transformative promise of the human genome for health and society.^1^ They anticipated genomics breakthroughs that could touch all of humanity and empower people to have longer healthier lives, ultimately creating a more prosperous society that also wields the human genome responsibly. Two decades on, human genomics now underpins the prospects of precision medicine and precision public health (Box 1). However, as further impactful advances increasingly depend on world-wide representation and collaboration, global-scale challenges that shackle progress must now be addressed. These are mainly economic, organizational, infrastructural, scientific, and Ethical, Legal and Social Implications (ELSI) challenges. Existing efforts lack coordination at the scale and scope required to overcome these challenges. In a time of heightened geopolitical uncertainty and legitimate citizen concern over the conscientious use of data and data-analysis methodologies, which include genomics big data and artificial intelligence (AI), we must draw inspiration from precedents where bold goals organized humanity to responsibly employ the best technologies and collaborative science toward their solution.^2^ In an era where intense corporate competition and geopolitical agendas threaten global collaboration, we must galvanize efforts to make precision medicine a universal gift — available and accessible to all of humanity for the generations to come.

## Box 1 Definitions of important terms.

**Precision medicine** is an approach to healthcare that takes into account inter-individual variability of genomic, environmental, and lifestyle characteristics. It aims to tailor medical treatment and prevention strategies to the specific characteristics of an individual or a group of individuals. This approach contrasts with the traditional one-size-fits-all model, where treatments and prevention strategies are developed based on population averages.

**Precision public health** applies the principles of precision medicine to the field of public health. It involves using advanced technologies, data analytics, and interventions to prevent disease, promote health, and improve healthcare delivery at the population level. Precision public health aims to identify and target interventions to specific vulnerable/disadvantaged groups and/or individuals who are at increased risk of disease or poor health outcomes, thereby maximizing the effectiveness and efficiency of public health efforts.

**Health span** refers to the period of a person’s life during which they are generally healthy and free from serious illness or chronic diseases. Unlike lifespan, which simply refers to the total length of an individual’s life, health span focuses specifically on the duration of time that a person enjoys good health and well-being. Maintaining and extending health span is a primary goal of public health efforts, medical interventions, and lifestyle choices aimed at promoting healthy aging and reducing the burden of chronic diseases and disability in older populations. Improving health span allows individuals to live longer, healthier, and more fulfilling lives.

The 20th century saw several remarkable research and development achievements brought about by a new way of doing big science.^3^ These pioneering megaprojects effectively synchronized efforts across government, industry, science, and engineering, rapidly achieving world-changing breakthroughs that brought humanity into the nuclear age and the space age. In the final quarter of the 20th century, such an integrated approach was adopted to launch the HGP. By the 1980s there was growing consensus that a global view of the human genome was necessary to unravel genetic links to disease, but the scale of the HGP was beyond the technological capabilities of the time.^2^ It thus took the first internationalized megaproject led by the International Human Genome Sequencing Consortium (IHGSC), mobilizing 20 centers across 6 countries, to invent and subsequently use the necessary technologies to complete the first draft of the human genome in 2003. Government, industry, science, and engineering collaborated effectively to usher in the age of genomics.

The exciting vistas the completed HGP offered quickly met a bigger dose of challenges. The first drafts of the human genome were limited by technologies of the time and were not truly complete; many sequences and features were still missing, as was the dimension of genetic diversity. Cultivating and scaling the fledgling field of genomics required not only improved sequencing technologies, but also cost falling by orders of magnitude. Thanks to grants announced by the US National Human Genome Research Institute (NHGRI) in 2004 to tackle these challenges, a burgeoning of massively parallel sequencing (MPS) technologies cut diploid genome sequencing costs faster than Moore’s Law predictions.^4^ With subsequent development of long-read technologies, greater dimensions of the human genome could be accurately and economically resolved. These advances empowered scientific consortia to sequence the first gap-less phased diploid genome to truly complete the HGP,^5^ assemble human pangenome references to better represent the breadth of genetic diversity,^6^ venture beyond single-nucleotide polymorphisms (SNPs) to catalog human genome structural variations (SVs), generate population genome baselines to discover the rarest alleles and employ multiple datasets to validate the clinical relevance of “variants of uncertain significance” (VUS).^7^ Genomics and biology blossomed, owing to these advanced sequencing technologies and their dramatic cost reduction. Government, industry, science, and engineering again tackled the problem together and created success.

Thanks to these bold integrative efforts, we could begin dissecting the interplay between our genes, environment, and lifestyle. Thus rose the field of precision medicine, in turn paving a path to precision public health. If the ultimate measure of impact for the human genome is the improvement of the global population health span (Box 1), then precision medicine and precision public health would be the means. Recent multidisciplinary developments have further facilitated precision medicine and precision public health.^8^ As genome sequencing costs start falling below $100, personal genomes are fast becoming widely affordable. As multi-omics technologies improve, researchers are moving beyond genome-wide association studies to multi-ome-wide association studies. As data science and AI find better application in life sciences, digitally-modeling complex human biology is getting easier, genetic disease screening programs grow in number and enrollment and, evidence for health economic benefits is accumulating.^9^ As precision medicine becomes a national priority in more countries, more governmental budgets and policies are being created to support its exploration.

Notwithstanding these encouraging trends, the impact of precision medicine on global population health span remains negligible thus far. Indeed, implementing global precision public health is an undertaking of unprecedented proportions; it requires equitable massive-scale participation, incorporation of large multi-omics datasets to expand the effectiveness of precision medicine, consensus health economic incentives and best practices, and responsible ELSI oversight. Several key global challenges must first be addressed if we want to scale up implementation and broaden impacts:

## Economic challenge

Global wealth inequality results in richer countries being abler to afford the expensive technologies, research programs, and population health initiatives needed for precision public health. In time, the richer countries move far ahead of “Low- and Middle-Income Countries” (LMICs) in terms of capabilities, knowledge, and progress. Left unchecked, precision medicine and precision public health become privileges reserved for rich countries; wealth inequality drives health inequality, contrary to our interconnectedness and interdependency as a species. Mounting evidence for the overwhelmingly positive health economics of precision public health makes it more pressing for LMICs to procure the necessary initial investment. Unless inequity is addressed, global precision public health will be hampered by limited participation and genetic representation, resulting in neither the rich nor the poor reaping maximum benefit.

## Organizational challenge

Asymmetries between countries, in terms of economic power and national intellectual capital, lead to great variance in their respective preparation and contribution to precision public health. As a result, national precision public health initiatives can range in implementation from the very advanced to the rudimentary to the non-existent. Also, as a result, their scientific designs can range from the pioneering to the copycats to the disjointed. Add geopolitics to the equation and we get a heterogeneous mixture of national initiatives that have limited collaboration with one another, despite humanity’s dependency on the quality of their output. Without an authoritative organizational framework that facilitates countries to share best practices and work together, global economic and intellectual resources for precision public health will continue being inefficiently deployed or even wasted.

## Infrastructural challenge

Considering inequity and disorganization, there is little surprise that the scientific, clinical, health financing, and ELSI works in various countries are still siloed. Precision public health depends on big data to power scientific discovery and clinical translation, as well as shared best practices on proof-of-value clinical programs and ELSI policies. To efficiently implement precision public health globally, data standards and implementation best practices must be broadly adopted to ensure interoperability. Common data security, analysis, sharing, and/or federation standards must also form the backbone of any data infrastructure supporting precision public health work. Silos must be avoided to the greatest extent possible if we want equity and efficient organization. A critical part of this will be prioritizing infrastructural elements that address security and trust.

## Scientific challenge

A useful interpretation of the genome for all of humanity requires a human pangenome reference comprised of many reference genomes from genetically diverse populations; genetic representation is critical for precision medicine. Although a Human Pangenome Project has been initiated, it requires much greater contribution from diverse populations if we are to more fully catalog human genetic variation, validate their clinical relevance, and illuminate VUS. Beyond our genome, incorporating multi-omics into large cohorts across diverse populations is the next frontier of translational discovery for precision medicine. Innovative technology and interoperable population multi-omics studies would be necessary for bringing the personal multi-ome into global standard clinical practice. These challenges necessitate global collaborative research frameworks. Global “team science” avoids redundancy of effort, promotes interoperability, brings knowledge equity, and enhances the translational output of key consortia. To align with precision public health goals, collaborative research must measure success in terms of contribution to public health outcomes.

## ELSI challenge

The Universal Declaration on the Human Genome and Human Rights (UNESCO) qualifies the human genome as a “heritage of humankind”. As such, ethical issues related to infringements of personal genomic data security, privacy and confidentiality, inequity of healthcare, selective inclusion in or exclusion from databases, discrimination in insurance or employment as well as overlooking individual autonomy on personal data remain major challenges. Genomic research should benefit all, including disadvantaged populations globally; yet, there are areas where capacity building and the ability to perform translational work remain a logistic or resource challenge. As social and economic inequities exist on a global scale, continued ELSI research among diverse cultures and indigenous populations around the world is needed to ensure that precision public health initiatives adapt sensitively in implementation.

The scale and scope of these challenges are daunting, even compared to those the HGP faced in its time. Nevertheless, the challenges must be met if we are to improve global human health span. Therefore, inspired by the precedents for success set by the bold integrative efforts of the past 80 years, we propose initiating the **Human Genome Project II (HGP2), a global precision public health project** to carry forth the legacy of the HGP and fulfill the promise to improve the human condition through deeper understanding of the human genome.

## HGP2

The mission of HGP2 will be to empower all of humanity to read and use the knowledge of their genomes to lead healthier and longer lives. HGP2 will set initial goals covering data generation, precision intervention, and clinical translation:

### Data generation goals

- Sequence the genomes of > 1% of the world population with maximal genetic representation (80 M individuals from > 100 countries);
- Contribute 50,000 telomere-to-telomere (T2T) phased diploid reference genomes from > 20 countries to a Human Pangenome Project and expand the pangenome reference to cover most diversity;
- Define standards and methodologies for integrating multi-omics into precision medicine, and;
- Create large multi-omics cohorts totaling > 0.1% of the world population from diverse populations and collect their multi-omics data (8 M individuals from > 10 countries).

### Precision intervention goals

- Define clinically actionable reporting and intervention best practices for carrier screening, incidental findings, dominant disorders, rare disease diagnosis, and pharmacogenomics;
- Implement clinically actionable reporting and intervention for all genomes sequenced within the scope of HGP2, and;
- Implement standardized health economics studies incorporating all genomes sequenced within the scope of HGP2, quantifying cost-effectiveness of genome-guided interventions.

### Clinical translation goals

- Catalog all genetic and multi-omic variation from genomes sequenced in HGP2;
- Clarify the clinical relevance of all genetic and multi-omic variation cataloged, and;
- Embed genomic and multi-omic findings into standard clinical practice and precision public health.

To achieve these goals, HGP2 is proposed to be organized to directly address key global challenges for precision public health:

### Addressing economic challenge

HGP2 is proposed to be supported by an open industry pledge, where any company with relevant technologies or solutions participate by pledging low costs for the scale of HGP2’s goals. We propose a commitment to a $99 or less re-sequenced genome for HGP2, as well as significant in-kind contribution toward 1000 de novo T2T reference assemblies for each participating national initiative. HGP2 will organize an international funding initiative or institution to be a “World Bank” for LMICs participating in HGP2. These measures address global inequity, leveling the economic playing field for countries so that all may bring the scale and representation needed for the HGP2 effort. A health economics work stream will be established to integrate, assess, and share global study experiences. This will support HGP2’s participating countries with their health economics analysis and simulations, informing them on economic feasibility and value proposition. Most funding for HGP2 will come from existing and future national initiative budgets, which will be deployed more efficiently and create more value within the HGP2 framework. HGP2 will also work with international bodies such as the World Health Organization (WHO) and United Nations Sustainable Development Goals (UNSDGs) for funding support.

### Addressing organizational challenge

As HGP2 is a global endeavor touching all of humanity, a national initiative participatory framework will help countries to coalesce around their common cause. This framework will be a national blueprint for precision public health, as well as provide access to HGP2 industry support, funding aid, standards, best practices, and scientific leadership. It will be equitable, interoperable, and public health outcomes oriented. Most of all, the HGP2 participatory framework will allow national initiatives to partake in something greater; through the HGP2 platform, national initiatives concurrently build a better country and a better world. Both ongoing and new national initiatives may join HGP2 through this participatory framework, in grassroot fashion. A global ELSI work stream comprised of representatives from participating countries or organizations will be established, pioneering new guidelines to preemptively protect the individual and serve as a watchdog for the responsible ethical use of data throughout HGP2.

### Addressing infrastructural challenge

Global Alliance for Genomics and Health (GA4GH) is a choice platform to bridge the infrastructural challenge with its many interoperability standards, work streams, and driver projects underway. HGP2 may join the GA4GH community as a premier driver project and utilize their data interoperability, federation, analysis, and security standards. HGP2 could contribute to existing GA4GH work streams and other driver projects, as well as create new ones with GA4GH, particularly in data security. With the GA4GH backbone and community, the HGP2 national initiative participatory framework will better break down regional silos, enhance global collaboration, and translate global scientific discovery to public health outcomes.

### Addressing scientific challenge

HGP2 will create a global research alliance to achieve its translational scientific goals and to foster knowledge equity among national initiative scientific strategies. Redundant effort must be avoided, when possible, to provide maximal support for existing scientific initiatives; this research alliance will foster “team science”, not competition. The HGP2 global research alliance will provide a participatory framework for scientific initiatives to access more interoperable data and resources to achieve their aims, in solidarity with the HGP2 mission.

### Addressing ELSI challenge

HGP2 stakeholders will engage ELSI experts to develop culturally sensitive, respectful to human rights and, legally sound approaches for all humanity. Training core expertise in genomics and counselling — such as clinical geneticists, counsellors, and social workers — will be prioritized. Working with governments, regional groups, patient representative groups and genomics organizations will be of immense importance. International bodies such as WHO and UNESCO could be partners to ensure that ELSI are incorporated into government policies. Public announcements and universal declarations on these issues must be made by these august bodies, so that they become official policies in all member countries. Hence, similar to the original HGP, a specific budget will be allocated to address these ELSI challenges to ensure their solution.

## Conclusion

Upon completion of the HGP in 2003, Francis Collins wrote, “For this grand vision (genomics benefiting humanity) to come true, however, we in the biologic research community need to pursue the next generation of research projects with the same determination and creativity that the dedicated scientists of the HGP used to spell out the human genetic code…We call on leaders across science and society, across academia and industry, and across political and geographic boundaries to join us on this exciting voyage to understanding ourselves.^1^” More than 20 years after the HGP’s completion, it is now time for HGP2 to deliver to humanity the promise of the human genome. We hope that this proposition inspires exemplary national initiatives to soon organize and collaborate around HGP2’s principles. HGP2 certainly will not stop at 1% of the world population; however, in achieving the goals set for the first 1%, we believe that HGP2 will have initiated a permanent paradigm shift toward precision public health globally. This will open the gates for the rest of humanity to use their genome to lead healthier and longer lives, fulfilling the vision of the HGP.
